# Patient experiences in trials of minimally invasive thoracic surgery: A mixed-methods study

**DOI:** 10.1007/s11701-026-03362-0

**Published:** 2026-04-01

**Authors:** Jennifer Donnelly, A. Brook, G. Wright

**Affiliations:** 1 Department of Cardiothoracic Surgery, St. Vincent’s Hospital Melbourne, Fitzroy, VIC Australia; 2 Independent Researcher, Mineola, TX USA; 3 Division of Cancer Surgery, Peter MacCallum Cancer Centre, Melbourne, Australia; 4 Research and Education Lead for Lung Cancer, Victorian Comprehensive Cancer Centre, Melbourne, Australia; 5 Department of Allied Health, Swinburne University of Technology, John Street, Hawthorn, VIC 3122 Australia

**Keywords:** Robotic-assisted surgery, Video-assisted thoracic surgery (VATS), Lung cancer surgery, Patient-reported outcomes, Clinical trials, Surgical innovation

## Abstract

**Background:**

Minimally invasive thoracic surgery, including robotic-assisted and video-assisted approaches, is increasingly used to treat lung cancer. While technical outcomes are well documented, patient experiences within clinical trials remain under-explored.

**Objective:**

To evaluate patient experiences of clinical trial participation involving advanced surgical modalities for lung cancer.

**Methods:**

This two-site, mixed-methods study evaluated patient perceptions of participation in the DURATION (ctDNA monitoring) and VGT-309 (fluorescent imaging) trials. Twenty-six participants completed quantitative surveys assessing pre-trial expectations, experiences, and collaboration with research teams. Sixteen participants completed semi-structured interviews, analyzed using thematic analysis.

**Results:**

Twenty-six participants completed surveys and sixteen completed interviews. Motivations for enrollment included advancing science (100%), altruism (80.8%), and personal benefit (61.5%). Satisfaction was universally high, with participants reporting positive surgical experiences, supportive staff interactions, and minimal procedural burden. Most participants (88.5%) expressed willingness to participate in future trials, and 96.2% would recommend trial involvement to others. Qualitative analysis identified seven domains characterizing patient experiences throughout the trial process: motivations for enrollment, communication from the trials team, influences on surgical decision-making, surgical expectations, postoperative care, challenges to participation, and overall experience. Challenges such as perioperative anxiety and logistical demands were minor and did not detract from overall satisfaction. Overall, participants described the trial experience as seamless and personally meaningful.

**Conclusion:**

Patients undergoing robotic-assisted or video-assisted thoracic surgery within clinical trials report overwhelmingly positive experiences. The integration of patient-centered communication and advanced technology creates a seamless perioperative journey. Clinicians should not hesitate to enroll appropriate patients in trials featuring surgical innovations.

**Supplementary Information:**

The online version contains supplementary material available at 10.1007/s11701-026-03362-0.

## Introduction

Lung cancer remains the leading cause of cancer-related mortality worldwide [1, 2]. The surgical management of pulmonary malignancies has undergone a significant paradigm shift since the introduction of the da Vinci surgical system (Intuitive Surgical, Sunnyvale, CA), with robotic-assisted thoracic surgery and video-assisted thoracic surgery emerging as standard minimally invasive approaches [3].

Robotic platforms offer technical advantages including tremor elimination, increased degrees of freedom, superior surgeon ergonomics, and enhanced three-dimensional visualization [4]. These advantages translate to clinical benefits: large-scale database analyses and systematic reviews have demonstrated that robotic-assisted lobectomy is associated with lower perioperative morbidity, reduced 30-day mortality, and fewer early postoperative complications compared to open thoracotomy, with comparable long-term survival [5, 6].

The present study drew participants from two clinical trials investigating advanced monitoring and imaging technologies:

- *The DURATION Trial*: This protocol uses droplet digital PCR to track circulating tumor DNA (ctDNA) in patients’ blood, enabling detection of molecular signs of cancer recurrence after minimally invasive lung cancer resection.
- *The VGT-309 Trials*: These protocols evaluate abenacianine, an imaging agent that, when injected before surgery, causes tumor cells to fluoresce under near-infrared light, aiding localization and more precise identification of cancer margins.

While the safety and feasibility of robotic platforms and established imaging adjuncts are well-documented [7], patient experiences within complex trial frameworks remain under-explored. Prior studies of patient experiences in experimental oncology trials suggest that decision-making is often influenced by a strong desire for personal benefit, leading patients to emphasise potential gains over risks; many participants also express a need for clearer, simplified information to better understand the trial’s primary scientific purpose versus its clinical limitations [8]. A recent meta-synthesis of 45 qualitative studies examining the cancer clinical trial journey identified informational needs, the desire to maintain hope, and practical and psychophysical burdens as recurring themes, yet noted that patient narratives from the post-trial period remain underrepresented in the literature [9]. A mixed-methods study of cancer drug trial participants at a major Australian cancer centre found that while patients reported generally positive experiences, communication challenges — particularly around written consent materials — emerged as a consistent concern across all stages of trial participation [10].

Research into patient perceptions of surgical innovation remains remarkably sparse. While many patients associate robotic-assisted platforms with advanced precision and faster recovery, these views are often shaped more by marketing language than by clinical evidence [11]. Prior research also indicates that patients may harbour anxieties about robotic surgery, including concerns about equipment reliability and reduced surgeon involvement [11–13]. Understanding how these perceptions influence trial participation remains important for optimising recruitment. To date, no study has examined patient experiences at the intersection of surgical innovation and clinical trial participation — specifically, how patients perceive being enrolled in a clinical trial when the trial involves advanced surgical modalities for a life-threatening illness.

This study aims to evaluate patient experiences of robotic-assisted and video-assisted thoracic surgery within clinical trial settings. The question addressed here — which has received limited attention in the medical literature — is how patients perceive being on a clinical trial when they have a life-threatening illness and are being treated with advanced surgical modalities. The answer will be of interest to physicians deciding whether to refer patients to clinical trials, and to those wishing to conduct trials involving life-threatening illnesses and advanced surgical techniques.

## Methods

### Study design and ethical oversight

This mixed-methods study combined quantitative survey data with qualitative insights from semi-structured interviews to develop a nuanced understanding of the patient experience. The study protocol received institutional review board approval from the St. Vincent’s Hospital Melbourne Research Ethics Committee (Approval No. 086/25). The research team included three members who have roles across academia, thoracic surgery, and clinical trial coordination with expertise in both qualitative and quantitative methods.

## Setting and participant selection

The recruitment base comprised patients enrolled in the *DURATION* (ddPCR; HREC 168.18) and *VERGENT* (VGT-309; HREC 051.21 and VGT-309 2B; HREC 076.24) trials who underwent either robotic-assisted or video-assisted thoracic surgery (VATS) at St. Vincent’s Hospital Melbourne or St. Vincent’s Private Hospital Melbourne. To be eligible, participants were required to be 18 years of age or older and proficient in English.

## Recruitment and consent

Participant recruitment was conducted over the months of August and September 2025. Eligible participants were initially contacted via telephone to gauge interest, followed by an email containing a link to a digital survey with an embedded information and consent form. For the qualitative component, purposive sampling was employed to ensure a diverse representation of age, sociodemographic status, and gender to reduce confounding factors. These participants were invited via telephone for a semi-structured interview, with formal consent materials provided electronically or via post. Interviews were scheduled at the participant’s convenience and conducted face-to-face, via telephone, or through videoconferencing.

## Data collection

### Quantitative Survey

An estimated 10–15 min patient experience survey was developed in REDCap, informed by established frameworks for assessing the research participant experience [14]. The instrument explored patient perspectives across three domains: (1) pre-trial expectations, (2) peri-trial experiences, and (3) perceived collaboration with the research and healthcare teams (Supplement 1).

### Qualitative Interviews

One-on-one semi-structured interviews (approximately 15–20 min) were completed in the months of September and October 2025 and audio-recorded. An interview guide with open-ended questions was developed to explore participant experiences in depth (Supplement 2). Participants were given the opportunity to review their responses prior to analysis; no changes were requested. To reduce social desirability bias, interviews were conducted by a researcher (JD) who was not directly involved in the participants’ clinical care. Participants were assured that their responses would be de-identified and would not affect their ongoing care. The interview guide included questions explicitly eliciting negative experiences and challenges (Supplement 2).

### Data analysis

#### Quantitative Analysis

Survey data were exported from REDCap and analyzed using descriptive statistics in SPSS Statistics version 31.0.1 [15].

#### Qualitative Analysis

Audio recordings were transcribed verbatim and analyzed using thematic analysis [16]. Two researchers (JD and AB) independently coded the data in ATLAS.ti Web [17]. Themes and subthemes were developed through discussion until consensus was reached. This independent process was maintained until a final consensus was reached on the thematic framework, ensuring methodological rigor and inter-rater reliability. Saturation was not formally assessed; however, the sample size of 16 interviews provided sufficient depth to identify recurring patterns across participants. This mixed method study was reported in accordance with the Good Reporting of a Mixed Method Study (GRAMMS) checklist [18].

## Results

### Participant characteristics

Of the eligible participant pool, 9/45 (20%) were not contactable and two declined participation with no reason given. A total of 26/33 (79%) of participants who agreed to receive the survey, completed the quantitative survey. The cohort was nearly evenly distributed by gender (53.8% women, 46.2% men) and primarily represented an older demographic, with 65.4% of participants aged 65 years or older. Most participants were retired (*n* = 17, 65.4%), while 8 (30.8%) remained in the workforce. The majority of the cohort (61.5%) had no prior experience with clinical trial participation.

Of the 25 participants invited for a semi-structured interview, 16 consented and completed the session (64.0% response rate). Among interview participants, 15/16 (94%) completed both the survey and the interview. Those who declined an interview, all completed the survey component of the study. The interview sub-cohort mirrored the broader survey population regarding gender (50.0% women) and age (62.5% aged > 65 years). Detailed participant demographics are provided in Table 1

**Table 1:** Characteristics of participants who completed a semi-structured interview (*n* = 16) or survey (*n* = 26)

Category	Semi-structured interviews *n* (%)	Survey *n* (%)
**Gender**		
Women	8/16 (50.0)	14/26 (53.8)
Men	8/16 (50.0)	12/26 (46.2)
**Age (years)**		
35–54	3/16 (18.8)	3/26 (11.5)
55–64	3/16 (18.8)	6/26 (23.1)
65–74	6/16 (37.5)	10/26 (38.5)
75+	4/16 (25.0)	7/26 (26.9)
**Employment Type**		
Employed/Self-Employed	5/16 (31.3)	8/26 (30.8)
Retired	10/16 (62.5)	17/26 (65.4)
Other/Unemployed	1/16 (6.2)	1/26 (3.8)

## Quantitative survey results

Survey results are organized into three key areas: enrollment motivations, satisfaction and clinical outcomes, and future participation intentions.

### Trial enrollment and motivations

Participants reported high levels of satisfaction with the pre-trial enrollment process; 100% agreed they received all necessary information and felt comfortable asking questions prior to consent. Primary motivations for enrollment (respondents could select multiple) included:

- Scientific Advancement: 100% aimed to help advance science for lung cancer treatment.
- Altruism: 80.8% sought to help future patients suffering from lung cancer.
- Personal Benefit: 61.5% hoped to obtain superior treatment for their own condition.

### Satisfaction and clinical outcomes

Overall satisfaction with the clinical trial was 100%. Specific high-satisfaction metrics included the surgical experience (100%), care provided by hospital-based trial staff (96.2%), and information regarding post-trial support (84.7%). Participants perceived the trial as both beneficial and low burden. 96.1% felt they had contributed meaningfully to medical research, and 84.6% reported an improved quality of life. Furthermore, 84.7% of participants disagreed that the trial was time-consuming or interfered with their daily routines.

### Perceptions of minimally invasive surgery

Participants held favourable perceptions of minimally invasive surgery compared with standard surgery. The majority agreed or strongly agreed that minimally invasive surgery is performed through smaller incisions (25/26, 96.2%), offers enhanced surgical precision (22/26, 84.6%), provides quicker recovery (21/26, 80.8%), carries a lower risk of complications (20/25, 80.0%), delivers improved surgical outcomes (20/26, 76.9%), and causes less pain (19/26, 73.1%). No participants disagreed with any item; remaining responses were neutral.

### Future participation and recommendations

Engagement levels remained high regarding future research; 88.5% of respondents indicated a willingness to participate in a future randomized clinical trial (RCT) should there be no clear “gold standard” treatment. Additionally, 96.2% stated they would recommend participation in this specific study or future trials conducted by this research team.

### Qualitative interview results

Thematic analysis of the semi-structured interviews yielded fifteen themes and seven sub-themes organized across seven core domains. These findings provide a deeper understanding of the survey data, detailing the nuanced experiences and perceptions of the participants. A summary of these domains, themes, and sub-themes, accompanied by illustrative participant quotes, is presented in Table 2. Detailed findings for each domain are presented below.

**Table 2:** Qualitative domains, themes, and representative quotes regarding patient experiences in minimally invasive thoracic surgery clinical trials

Theme	Subtheme	Representative Quote
**Domain 1: Motivations and Influences Regarding Enrollment**		
**Altruistic Motivations**	Desire to contribute to medical knowledge	“You feel good. You’re helping other people.” (ID06)
**Expectation of Superior Care**	Perceived advantage of specialized oversight	“The doctor comes, or the trial people come, and you sort of felt at ease because … you had a lot of people looking after you.” (ID03)
**Risk-Benefit Appraisal**	Personal health goals vs. trial objectives	“I was very comfortable going along with it. It was just hopefully a tool that would aid the surgeon and I was all for that.” (ID10)
**Foundational Trust**	Confidence in study protocol and surgical expertise	“If somebody talks to me about a trial and it seems to me that it makes sense, then I’ll definitely do it.” (ID01)
**Domain 2: Communication from the Clinical Trials Team**		
**Protocol and Consent Clarity**	Transparency of study procedures	“They sort of explained everything really well.” (ID03)
**Staff Attentiveness and Empathy**	Reassurance through clinical team rapport	“I just remember the clinical trial staff as being very thorough and pleasant … they were certainly reassuring.” (ID04)
**Domain 3: Influences on the Decision to Undergo Surgery**		
**Surgeon-Related Influences**	Trust in the surgeon’s expertise	“I felt 100% confident with [surgeon’s name].” (ID07)
	Referring doctors’ endorsement of the surgeon	“[Surgeon’s name] was referred to me by my own personal doctor out here who knew him.” (ID07)
	Surgeon’s communication style and explanations boosted confidence	“The trial was explained to me very clearly … I was very comfortable going along with it.” (ID10)
**Perceptions of Surgical Modality and Outcomes**	Perceived procedural advantages (e.g., precision, rapid recovery)	“[It’s] very precise, so I was sort of with ease after that.” (ID03) “It would be a lot more steady for the surgeon. There’s no shaking, there’s no ifs and buts, it’s very clean in and out.” (ID06)
**Domain 4: Expectations of Surgery within the Clinical Trial**		
**Congruence of Surgical Expectations and Clinical Outcomes**	Perceived clinical benefits of surgical resection	“It wasn’t just the central lobe, which was the prediction, but it was something in the lower lobe … They discovered extra areas they needed to take out.” (ID14)
	Positive recovery trajectory and minimal procedural burden	“Normally after these robotic surgeries, they’re all fine. I recover very quickly.” (ID01) “Because it’s less invasive … you’re not sliced open so much.” (ID02) “You’re going and get a lung operation to get cancer removed overnight and come home. There’s not much to complain about … It’s a bit of a no-brainer.” (ID06)
**Domain 5: Postoperative Care and Follow-up**		
**Continuity of Support**	Accessibility of trial-specific support staff	“I had numbers to ring, but I didn’t need to talk to anyone to tell you the truth.” (ID07) “Really positive. I felt like I was in really good hands. I had strong communication. [The clinical trials nurse] gave me her mobile number and got in contact, you know, frequently post that meeting as well as to next steps.” (ID11)
**Adequacy of Follow-up Protocols**	Perceived adequacy of postoperative monitoring	“Afterwards … blood tests and the ECGs and then … a couple of phone calls after. So, it wasn’t very, it wasn’t very involved.” (ID02)
**Domain 6: Challenges to Clinical Trial Participation**		
**Perioperative Anxiety Management**	Normalization of perioperative apprehension	“I would understand nervousness … it wasn’t long before I was back to normal again.” (ID16)
**Logistical and Time Commitments**	Minimal impact of trial-related time commitments	“I understand it’s to help other people as well as myself. I was treated really well … I went back quite a few times with blood tests done and testing the heart … and [it] wasn’t really any interruption in my time.” (ID06)
**Domain 7: Overall Experience**		
**Perceived Continuity and “Seamlessness”**	Perceived ease of the surgical journey	“It was very seamless.” (ID10) “It was a really easy, positive experience.” (ID13) “I can’t believe I had some of my lung taken out, and I can’t tell … You can hardly see the scars. The little things. It’s just unbelievable.” (ID07)
**Propensity for Future Participation and Advocacy**	Willingness to advocate for clinical research	“I would say give it, give it a go… my experience… it wasn’t long before I was back to normal again.” (ID16) “If you can get more advanced medical care, definitely.” (ID06)

Note: ID, participant identification number; ECG, electrocardiogram. Brackets [ ] indicate text added or modified for clarity or de-identification. Ellipses (…) indicate omitted text for brevity

### DOMAIN 1: motivations and influences regarding enrollment

Participants were motivated to enroll by a convergence of altruistic intent and personal benefit, viewing trial participation as both a contribution to medical progress and an opportunity to access specialized care. These motivations were reinforced by foundational trust in the research protocol, surgical team, and institution. A prominent driver was the desire to advance medical science and help future patients. Participants viewed their involvement as personally meaningful even while facing a life-threatening diagnosis, with one noting: “I just thought it was important to me personally… to get the cancer removed for a start, but… to understand that I’m helping in some small way, helping advance a bit of science.” (ID12).

Beyond altruism, participants anticipated that trial enrollment would provide access to enhanced medical attention, describing it as an opportunity to receive “the most advanced thing at the moment, which means I’m getting the best of care” (ID06). The perception of low risk and high potential benefit made enrollment appear both logical and appealing. Foundational trust was critical. Endorsements from referring physicians played a significant role, as did the surgeon’s communication style. Trust extended to the institution itself, with participants expressing confidence that any trial conducted within the hospital would be managed with appropriate clinical rigor.

### DOMAIN 2: communication from the clinical trials team

Participants consistently described interactions with clinical trial staff as clear, thorough, and supportive. Information regarding the study protocol and consent process was communicated transparently, with participants receiving detailed written materials and being encouraged to consult with family members and personal physicians before providing consent. Interactions with staff were characterized by professionalism, empathy, and attentiveness. Even those with minimal contact found reassurance in knowing support was readily accessible. Overall, participants described their engagement with the clinical trials team as aligning with expectations for pleasant, supportive, and professional care.

### DOMAIN 3: influences on the decision to undergo surgery

The decision to undergo minimally invasive surgery was shaped by three factors: trust in the surgeon’s expertise, endorsements from referring physicians, and favorable perceptions of the surgical modality. Trust in the surgeon was central. Participants emphasized the importance of the surgeon’s specialized skills, with professional endorsement from referring doctors providing critical reassurance. One participant explained: “[Surgeon’s name] was referred to me by my own personal doctor out here who knew him, and I just did not have any concerns at all about what he was going to do with robotics” (ID07).

Robotic-assisted and video-assisted surgery were widely viewed as offering superior precision and outcomes compared to open surgery. Participants anticipated faster recovery and minimal physical burden, with one characterizing it as “a bit of a no-brainer” (ID06). These perceptions were reinforced by independent research, testimonials, and confidence in Australia’s healthcare safety standards.

### DOMAIN 4: expectations of surgery within the clinical trial

Participants’ expectations were largely met or exceeded, with many reporting outcomes that surpassed initial anticipations. Recovery was characterized by short hospital stays, minimal pain, and rapid return to normal activities. One participant reflected: “I can’t believe I had some of my lung taken out, and I can’t tell… you can hardly see the scars… It’s just unbelievable” (ID07).

Participants identified specific clinical benefits from trial participation, particularly regarding tumor detection. Several reported that more lesions were identified and resected than anticipated based on preoperative imaging: “It lights up the tumour and there’s this nice big bright green spot and he said we got that and by the way we’ve got two others as well that they found” (ID01). Overall, the trial framework was seen as enhancing rather than complicating the surgical experience.

### DOMAIN 5: postoperative care and follow-up

Postoperative care was described as straightforward and supportive. Having direct access to clinical trial staff provided significant reassurance, regardless of whether participants actively utilized this support. The variability in follow-up intensity reflected individual needs, with participants expressing satisfaction that support was appropriately tailored to their recovery trajectories.

Follow-up comprised standard surgical procedures including reviews with the surgeon and calls from trial coordinators. This low burden was viewed positively, as trial-related requirements did not impose significant demands beyond standard postoperative care. Minor complications were perceived as resolving over time and within expectations previously outlined by the surgical team.

### DOMAIN 6: challenges to clinical trial participation

Challenges were characterized as minor inconveniences rather than significant barriers. Perioperative anxiety was acknowledged but viewed as a standard aspect of undergoing surgery rather than trial-specific. Confidence in the surgical team and the minimally invasive approach mitigated much of this anxiety.

Logistical demands posed only minor hurdles. One participant described the pre-surgical day as “tedious” but “not difficult” (ID01). Time commitments were generally perceived as manageable and justified by anticipated benefits. Hospital parking was mentioned by some as a minor inconvenience but not a substantial barrier.

### DOMAIN 7: overall experience

Participants reported an overwhelmingly positive experience, frequently characterizing the process as straightforward, seamless, and personally meaningful. The integration of trial procedures into standard surgical care was found unobtrusive and well-coordinated. All participants indicated willingness to enroll in future clinical trials, provided the protocol is appropriate and scientifically sound. Responses regarding recommending trial participation were uniformly positive, though participants emphasized the importance of critically evaluating information before consenting. Altruism emerged as a sustained motivator throughout, with participants expressing satisfaction that their involvement could benefit future patients.

#### Mixed-methods integration

Table 3 presents a joint display mapping key quantitative findings to their corresponding qualitative domains, demonstrating where and how the quantitative and qualitative strands of the study were integrated. Across all seven domains, quantitative survey findings were consistent with the qualitative themes identified through semi-structured interviews, with the qualitative data providing contextual depth to the patterns observed in the survey responses.

**Table 3:** Joint display of quantitative survey findings and corresponding qualitative domains

Quantitative Finding	Related Qualitative Domain	Representative Theme
100% aimed to advance science; 80.8% motivated by altruism; 61.5% hoped for better treatment	Domain 1: Motivations and Influences Regarding Enrollment	Altruistic motivations; expectation of superior care; risk–benefit appraisal
100% satisfied with pre-trial information; 100% comfortable asking questions	Domain 2: Communication from the Clinical Trials Team	Protocol and consent clarity; staff attentiveness and empathy
96.2% agreed minimally invasive surgery involves smaller incisions; 84.6% agreed it offers enhanced precision; 80.8% agreed it provides quicker recovery	Domain 3: Influences on the Decision to Undergo Surgery; Domain 4: Expectations of Surgery within the Clinical Trial	Perceptions of surgical modality and outcomes; congruence of surgical expectations and clinical outcomes
96.2% satisfied with hospital-based trial staff; 84.7% satisfied with post-trial support information	Domain 5: Postoperative Care and Follow-up	Continuity of support; adequacy of follow-up protocols
84.7% disagreed trial was time-consuming; 73.1% disagreed trial caused anxiety	Domain 6: Challenges to Clinical Trial Participation	Perioperative anxiety management; logistical and time commitments perceived as minimal
88.5% willing to participate in future RCT; 96.2% would recommend trial participation	Domain 7: Overall Experience	Perceived seamlessness; propensity for future participation and advocacy

## Discussion

This mixed-methods study explored the experiences of participants enrolled in clinical trials involving *robotic-assisted surgery or VATS* across two tertiary centers in Melbourne, Australia. To our knowledge, this is the first study to examine patient satisfaction when advanced surgical modalities are combined with clinical research protocols. The findings demonstrate that patients are highly satisfied with trial participation because the experience directly aligns with their primary motivations for enrolling: advancing science, altruism, and personal benefit.

Participant satisfaction was underpinned by three factors:

First, the desire for better treatment was met by deep trust in the surgeon’s expertise and the perceived precision of fluorescence-guided surgery. The use of fluorescent imaging acted as a “technological GPS,” reassuring patients that their tumors were being identified and resected with more accuracy than the naked eye. Structured pre-operative education and transparent communication were pivotal in fostering this confidence, aligning with previous research highlighting the positive impact of clear information on patient perceptions in robotic-assisted surgery [11], other surgical contexts [19, 20], and clinical trials broadly [21–23].

Second, participants appreciated the rigorous surveillance inherent to trial protocols. In the *DURATION study*, knowing their progress was being monitored more frequently than in standard care provided a sense of security. This perception that trial participation offers superior care is consistent with prior research [22, 24, 25].

Third, the altruistic goal of helping others—consistent with prior research on trial motivations [25]—was supported by an experience participants frequently characterized as “*seamless*”. Minimal surgical discomfort and faster recovery through minimally invasive approaches allowed participants to contribute to medical progress without undue physical burden.

These results suggest that concerns about patient burden should not deter physicians from offering enrolment in appropriate trials featuring novel technology. The perceived risk of the “unknown” is outweighed by patients’ desire to advance science and the reassurance provided by an empathetic clinical trials team. Future studies should examine whether these findings generalise to other surgical specialties, diverse patient populations, and different types of technological innovation.

While participants expressed confidence in the surgical technology, the qualitative findings highlight that trust in the surgical team, clear communication from clinical trials staff, and supportive postoperative care were consistently emphasised as key contributors to satisfaction. Attributing satisfaction primarily to technology would oversimplify the patient experience. Across the seven domains, participants repeatedly attributed their positive experience to interpersonal factors — trust in the surgeon (Domain 3), quality of communication from the trials team (Domain 2), and supportive postoperative care (Domain 5) — alongside, rather than secondary to, their perceptions of the technology itself. Notably, neither of the researchers who conducted interviews and data analysis (JD and AB) were involved in the direct clinical care of participants, which strengthens the credibility of these findings.

### Strengths and limitations

A primary strength of this study is the mixed-methods design, which combined quantitative survey data with qualitative interviews to yield a nuanced understanding of patient experiences. The qualitative component provided deep insight into motivations, trust, communication, and perceptions of surgical innovation.

This study has several limitations.

First, the self-reported nature of both surveys and interviews may introduce social desirability bias, with participants potentially overstating satisfaction. Although the interviewer was not part of the direct clinical care team, participants’ awareness that the study was affiliated with their treating institution may have influenced their willingness to express negative views. This is an inherent limitation of institutional research on patient experience.

Second, the sample size (*n* = 26 survey; *n* = 16 interviews) and geographic limitation to a single institutional network in Melbourne, Australia, constrain the generalisability of these findings. The cohort was predominantly older, retired, and English-speaking, and may not reflect the experiences of younger, working, culturally diverse, or non-English-speaking populations. These findings should be considered hypothesis-generating rather than definitive, and multi-site studies with larger, more diverse samples are recommended.

Third, only participants who completed the trial were included in this study, which may introduce selection bias and lead to an overestimation of satisfaction. No patients withdrew from either trial. However, patients who declined trial participation at the recruitment stage may have had different perspectives, and their reasons for declining are not captured here. Future research could incorporate the views of patients who declined or withdrew from clinical trials to provide a more balanced understanding of the patient experience.

Fourth, several satisfaction indicators approached ceiling values. While consistent with prior research on clinical trial participant satisfaction (14, 24), the possibility of positively framed survey items, social desirability bias, and selection effects may contribute to these findings.

Fifth, the focus on lung cancer resections within specific trial protocols may not reflect experiences in other surgical specialties or trial designs.

Sixth, no Aboriginal or Torres Strait Islander participants were enrolled in either trial, which limits the generalisability of these findings to Indigenous Australians, who experience disproportionate lung cancer burden. Future research should develop culturally appropriate protocols to explore the clinical trial experiences of these communities.

## Conclusion

This study suggests that participants undergoing *robotic-assisted surgery or VATS* within a clinical trial framework report overwhelmingly positive experiences, often describing the perioperative journey as “*seamless*”. This high level of satisfaction is driven by a desire to advance scientific knowledge and a perception that trial participation offers superior clinical precision and rigorous monitoring.

These findings suggest that enrolling patients in appropriate clinical trials involving advanced surgical technology is well received by participants. Participants viewed technical innovation and trial-specific surveillance not as burdens, but as significant sources of reassurance and personal benefit. By integrating patient-centered communication with advanced surgical platforms, clinical trials can provide an experience that meets, and often exceeds, the expectations of patients navigating a life-threatening diagnosis.

## Supplementary Information

Below is the link to the electronic supplementary material.

Supplementary Material 1

Supplementary Material 2

Supplementary Material 3

## Data Availability

The data that support the findings of this study are available from the corresponding author, J.D., upon reasonable request.
