# Supplementary material for: Patient experiences in trials of minimally invasive thoracic surgery: A mixed-methods study
Source: J Robot Surg. 2026 Apr 1;20(1):387. doi: 10.1007/s11701-026-03362-0 (PMC13038631; doi:10.1007/s11701-026-03362-0)
Supplement: Supplementary file 1 — Supplementary Material 1 [file 11701_2026_3362_MOESM1_ESM.docx]

# Good Reporting of A Mixed Methods Study (GRAMMS) checklist

| **Guideline** | **Section: page** |
| --- | --- |
| Describe the justification for using a mixed methods approach to the research question | Methods |
| Describe the design in terms of the purpose, priority and sequence of methods | Methods |
| Describe each method in terms of sampling, data collection and analysis | Methods |
| Describe where integration has occurred, how it has occurred and who has participated in it | Results |
| Describe any limitation of one method associated with the present of the other method | Discussion – Strengths and Limitations section |
| Describe any insights gained from mixing or integrating methods | Discussion |

O'Cathain A, Murphy E, Nicholl J. The quality of mixed methods studies in health services research. J Health Serv Res Policy. 2008;13: 92-98.
