# Supplementary material for: Patient experiences in trials of minimally invasive thoracic surgery: A mixed-methods study
Source: J Robot Surg. 2026 Apr 1;20(1):387. doi: 10.1007/s11701-026-03362-0 (PMC13038631; doi:10.1007/s11701-026-03362-0)
Supplement: Supplementary file 2 — Supplementary Material 2 [file 11701_2026_3362_MOESM2_ESM.docx]

**Supplement 1 Survey**

# Clinical trial experiences of patients undergoing <insert surgery type>

You have been sent this survey as you have indicated your interest in completing a survey to explore your experiences on a clinical trial when undergoing <insert surgery type> thoracic surgery. To continue you simply need to sign the e-consent and continue answering the questions.

Thank you so much for considering this important research. Department of Cardiothoracic and Oncology Surgery St.Vincent's Hospital Melbourne


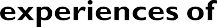

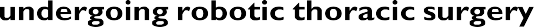


1. As a patient who has undergone robotic surgery and participated on a clinical trial at St. Vincent's Private Hospital, you are invited to participate in a study investigating your experiences on a clinical trial. If you would like to participate, please read the attached document below and then confirm your consent by providing a signature in the box below.

[Attachment: "PICF SVPHM_Participant Survey_RTS_v1.1_01052025_clean.pdf"]

1. I have read the Participant Information Sheet (pdf attached above) or someone has read it to me in a language that I understand. I understand the

purposes, procedures and risks of the research described in the project. I have had an opportunity

to ask questions and I am satisfied with the answers I have received. I freely agree to participate in this research project as described and understand that I am free to withdraw at any time during the project without affecting my future health care.

Participant's Name:

1. What best describes your gender? Male

. Female


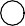

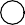

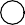

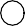


Non-binary/third gender Prefer Not to Say

1. What is your age range (in years)? 18-34


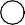

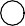

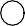

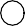

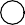


35-54

55-64

65-74

75+


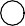

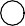

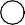


1. Have you participated on a clinical trial in the past? Yes No Unsure
2. What is your current employment status? Employed Unemployed Retired

Do not wish to answer


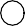

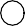

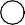

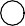


1.
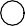

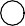

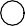

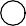

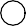
The information given to me before I joined the trial was everything I wanted to know (for example: visits and procedures, time commitment, who to contact with questions).


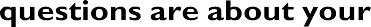

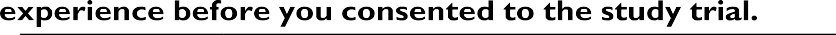


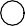

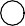

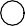

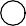

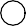
The information given to me before I joined the trial was easy for me to understand.


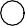

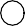

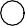

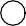

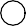
I felt comfortable that I could ask any questions before I joined the trial.


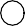

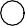

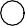

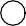

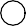
I understood the treatment process in the study (including potential side effects).

1. Through taking part in this study, I hoped to... Obtain better treatment for my lung cancer (select all answers that are relevant to you). Enhance my quality of life

Help advance science for the treatment of lung cancer


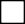

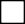

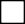


Help others who may suffer from lung cancer Obtain education about treatment/improving my health


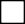

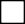


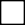
 The chance to receive better care from healthcare staff


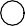

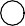

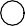

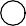

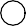
care provided by clinical trial staff whilst in hospital.


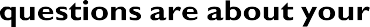

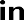


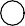

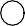

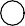

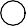

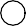
I was satisfied with the general interactions with clinical trials staff whilst in hospital.


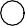

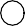

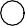

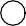

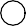

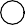

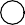

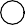

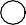

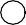
I felt comfortable asking questions to clinical trials staff in hospital.

I was satisfied with my

experience in undergoing robotic surgery.


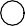

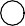

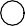

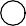

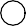
information I received about future support after the trial (for example: future treatment, follow up contact details).


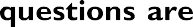

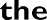


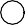

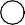

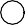

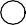

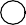
Overall, I was satisfied with my interactions with clinical trials staff after discharge.


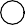

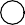

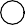

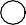

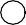
Overall, I was satisfied with my experience on a clinical trial.


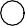

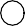

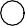

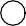

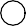
If given the opportunity, I would be willing to participate in a randomised clinical trial in the future if there is no clear choice for doctors to recommend one treatment over another.


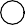

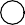

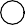

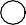

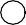
I would recommend participation in this clinical study or future clinical studies delivered by this team.


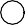

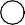

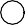

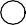

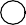
By participating in the clinical trial, I received enhanced medical care by being closely monitored by the research team

By participating in the clinical trial, I contributed to medical research, such as enhanced specialists' understanding of disease

By participating in the clinical trial, I was able to take a more active role in my own care

By participating in the clinical trial, the disease process was able to be better managed and I had an improved quality of life

By participating in the clinical trial, my surgical outcome was worse than if I had not participated

Participating in the clinical trial was time-consuming and interfered with my daily life or work commitments

By participating in the clinical trial, I incurred additional financial expenses

Participating in the clinical trial caused me anxiety and stress

Participating in the clinical trial made me concerned about the confidentiality and privacy of my personal health information

The following are questions around your perceptions of <insert surgery type> surgery

1. <insert surgery type> surgery is performed through small incisions than standard surgery

Strongly disagree

Disagree Neither agree or disagree

Agree Strongly agree

<insert surgery type> surgery causes less pain than standard surgery

There is a quicker recovery from <insert surgery type> surgery than from standard surgery

<insert surgery type> surgery has a lower risk of complications than standard surgery

<insert surgery type> surgery has enhanced surgical precision compared with standard surgery

<insert surgery type> surgery has improved surgical outcomes compared with standard surgery

| 9a | The following question is asking about you previous | Previous hospitalisation |
| --- | --- | --- |
|  | hospital encounters. Which of the following have you | Previous surgical admission |
|  | had (select all relevant): | Previous thoracic surgical admission |
|  |  | Previous robotic surgery |
|  |  | None of the above |
|  |  | The chance to receive better care from healthcare |
|  |  | staff |

9b How did this hospital encounter as a patient on a clinical trial compare to each hospital admission that

you have experienced e.g. previous hospitalisation, previous surgical admission, previous thoracic

surgical admission, previous <insert surgery type> surgery?

1. Based on your pre-admission expectations, what was the best thing about being on a clinical trial?
2. Based on your pre-admission expectations, what was the worst thing about being on a clinical trial?
3. Based on your pre-admission expectations, what was the best thing about having <insert surgery type> surgery?
4. Based on your pre-admission expectations, what was the worst thing about having <insert surgery type> surgery?

PRESS SUBMIT to submit your responses. Thank you for your participation in this survey.
