# Supplementary material for: Patient experiences in trials of minimally invasive thoracic surgery: A mixed-methods study
Source: J Robot Surg. 2026 Apr 1;20(1):387. doi: 10.1007/s11701-026-03362-0 (PMC13038631; doi:10.1007/s11701-026-03362-0)
Supplement: Supplementary file 3 — Supplementary Material 3 [file 11701_2026_3362_MOESM3_ESM.docx]

**Supplement 2** Semi-structured interview guide and inquiry logic

*Note: Identical interview guides were administered to each surgical cohort, with the procedure name (robotic-assisted surgery or VATS) inserted as appropriate. “<procedure performed>” denotes where the specific procedure name appeared.*

| **Demographic characteristics of participants** |
| --- |
| What is your age range? (years) |
| 18-34 |
| 35-54 |
| 55-64 |
| 65-74 |
| 75+ |
| What gender do you identify as? |
| What is your employment status?  Employed  Unemployed  Retired |

Prompts will be given to elicit more information if short answers are provided for the below e.g tell me more about that/this, can you please expand on that/this etc.

| **Interview Questions** | **Inquiry Logic** |
| --- | --- |
| Before your <procedure performed> how did you feel about being in a clinical trial? | Pre-trial perceptions and decision-making |
| Describe your interactions with clinical trials staff before you consented to the trial and at the time of consent. | Interactions with clinical trials staff. |
| How well did you feel you understood the information given to you about the clinical trial? | Confidence in participation |
| Describe the level of confidence you had about being on a clinical trial and having <procedure performed>. What factors contributed to increasing or decreasing your confidence e.g. trial protocols, surgical approach, medical team, risk/benefits? | Confidence in participation/perceived benefit |
| Thinking about your time in hospital, can describe your interactions with clinical trials staff? | Interactions with clinical trials staff. |
| How did their involvement affect your care? | Hospital experience |
| Thinking about your experience after discharge from hospital, describe your experiences recovery from <procedure performed> whilst on a clinical trial e.g. adequacy of follow up communication, consultations with clinical trials staff etc. | Post-surgery experience and follow up. |
| Reflecting on your experience on a clinical trial, what was the worst thing about being on a clinical trial? | Challenges |
| Reflecting on your experience on a clinical trial, what was the best thing about being on a clinical trial? | Benefits |
| Reflecting on your experience undergoing <procedure performed>, describe the worst thing about this experience? | Challenges |
| Reflecting on your experience undergoing <procedure performed>, describe the best thing about this experience? | Benefits |
| If given the choice again, describe why you would or wouldn’t consent to be a participant on a clinical trial? | Future considerations |
| Would you recommend being on a clinical trial to others? Why/Why not? | Future considerations and recommendations |
